# Spatio-temporal distribution of malaria and its association with climatic factors and vector-control interventions in two high-risk districts of Nepal

**DOI:** 10.1186/1475-2875-13-457

**Published:** 2014-11-25

**Authors:** Meghnath Dhimal, Robert B O’Hara, Ramchandra Karki, Garib D Thakur, Ulrich Kuch, Bodo Ahrens

**Affiliations:** Nepal Health Research Council (NHRC), Ministry of Health and Population Complex, Ramshah Path, Kathmandu, Nepal; Biodiversity and Climate Research Centre (BiK-F), Senckenberg Gesellschaft für Naturforschung, Frankfurt am Main, Germany; Institute for Atmospheric and Environmental Sciences (IAU), Goethe University, Frankfurt am Main, Germany; Institute of Occupational Medicine, Social Medicine and Environmental Medicine, Goethe University, Frankfurt am Main, Germany; Department of Hydrology and Meteorology (DHM), Ministry of Science, Technology and Environment, Babar Mahal, Kathmandu, Nepal; Ministry of Health and Population (MoHP), Government of Nepal, Ramshah Path, Kathmandu, Nepal

**Keywords:** Climate change, Cross-border, Hotspots, Imported malaria, Insecticide-treated nets, Indoor residual spraying, Jhapa, Minimum temperature, Morang, Relative humidity

## Abstract

**Background:**

Over the last decade, the incidence of confirmed malaria has declined significantly in Nepal. The aim of this paper is to assess the spatio-temporal distribution of malaria and its association with climatic factors and vector control interventions in two high-risk districts of Nepal.

**Methods:**

Hotspot analysis was used to visualize the spatio-temporal variation of malaria incidence over the years at village level and generalized additive mixed models were fitted to assess the association of malaria incidence with climatic variables and vector control interventions.

**Results:**

Opposing trends of malaria incidence were observed in two high-risk malaria districts of eastern and far-western Nepal after the introduction of long-lasting insecticidal nets (LLINs). The confirmed malaria incidence was reduced from 2.24 per 10,000 in 2007 to 0.31 per 10,000 population in 2011 in Morang district but increased from 3.38 to 8.29 per 10,000 population in Kailali district. Malaria hotspots persisted mostly in the same villages of Kailali district, whereas in Morang district malaria hotspots shifted to new villages after the introduction of LLINs. A 1° C increase in minimum and mean temperatures increased malaria incidence by 27% (RR =1.27, 95% CI =1.12-1.45) and 25% (RR =1.25, 95% CI =1.11-1.43), respectively. The reduction in malaria incidence was 25% per one unit increase of LLINs (RR =0.75, 95% CI =0.62-0.92). The incidence of malaria was 82% lower in Morang than in Kailali district (RR =0.18, 95% CI =0.11-0.33).

**Conclusions:**

The study findings suggest that LLIN coverage should be scaled up to entire districts rather than high-incidence foci only. Climatic factors should be considered for malaria micro-stratification, mosquito repellents should be prescribed for those living in forests, forest fringe and foothills and have regular visits to forests, and imported cases should be controlled by establishing fever check posts at border crossings.

**Electronic supplementary material:**

The online version of this article (doi:10.1186/1475-2875-13-457) contains supplementary material, which is available to authorized users.

## Background

Nepal has made significant progress in reducing the incidence of malaria by more than 84% over the last decade [1]. It has already achieved and exceeded the target of the Millennium Development Goals (MDGs) and universal coverage of malaria control interventions, and the Roll Back Malaria (RBM) targets of 2010 [1–5]. However, an increasing proportion of *Plasmodium falciparum* and imported malaria has been observed in the last decade [1]. Although the analysis of malaria data from 31 malaria-risk districts has shown a significant decline of confirmed malaria and *Plasmodium vivax* malaria incidences, no decline was found for *P. falciparum* and clinically suspected malaria incidences [1]. Malaria cases in Nepal are highly clustered and vector control interventions are not uniformly implemented. Accurate identification of malaria foci or clusters at the local level can greatly increase the effectiveness of vector control interventions whereas not identifying foci can cause effective control measures to fail [6]. Hence, an accurate mapping of malaria-endemic foci at village development committee (VDC) or household levels improves the effectiveness and cost-effectiveness of vector control interventions.

Although about 84% (23 million) of the people in Nepal were estimated to be at risk of malaria in 2012, with 4% at high-risk [7], the recent microstratification of malaria-risk areas in 2012 at the VDC level shows only approximately 13.02 million people (48%) living in malaria-endemic VDCs [2]. Out of the estimated total population living in endemic areas, 0.98 million (3.6%) live in high-risk VDCs, 2.7 million (9.8%) live in moderate-risk VDCs, and 9.4 million (34.5%) in low-risk VDCs. Similarly, there are fewer districts classified as high- and moderate-risk (25 instead of 31) and the overall population living in VDCs at risk (estimated at 1,254 VDCs out of 3,972) is declining [2], which indicates the progress made to eliminate malaria-endemic VDCs (foci) and to achieve the malaria elimination goal by 2026.

The high-risk areas consist of forest, forest fringe, foothills, river belts, hills, and river valleys. Malaria incidence has declined in districts regardless of whether vector control interventions (i.e., implementation of indoor residual spraying (IRS) and distribution of long-lasting insecticidal nets (LLINs)) have been implemented [1]. Moreover, reports of malaria have increased in high-risk districts (Kailali, Nawalparasi, Dhanusha, and Mahottari) where vector control interventions were in place [5]. The Global Fund to Fight AIDS, Tuberculosis and Malaria (GFATM) began supporting a malaria control programme in 13 high-risk districts in 2004 with support for rapid diagnostic test (RDT) kits, artemisinin combination therapy (ACT), LLINs and information, education and communication/behaviour change communication (IEC/BCC) for LLIN use [5]. The distribution of LLINs started in 2006 in both districts.

Climatic factors such as temperature, humidity and rainfall play important roles in malaria transmission [8]. Temperature is the major determinant of malaria risk [9], but rainfall is important where temperature is not a limiting factor (e.g., in tropical areas of Africa and Bangladesh) [10, 11]. Humidity is a direct product of temperature and rainfall and affects the distribution and longevity of vector mosquitoes, which in turn affects malaria transmission [8]. The effect of climate change on malaria transmission in temperate regions and tropical highlands has been reported in many recent studies [12–14].

The main aim of this study was to assess the spatio-temporal distribution of malaria and its association with climatic factors and vector control interventions in two high-risk districts of Nepal.

## Methods

### Study area

**Figure 1:**
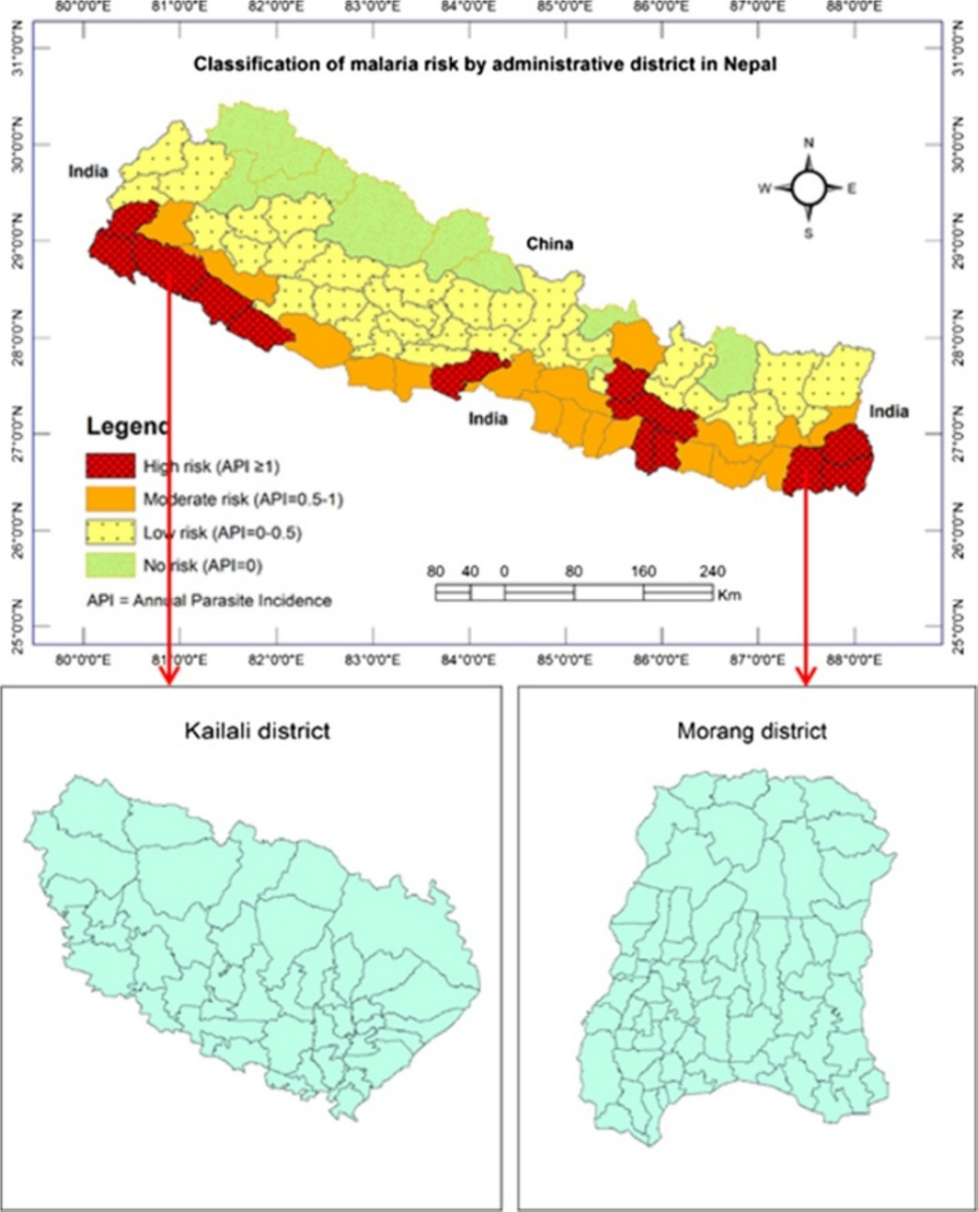
**Classification of malaria risk districts in Nepal and study areas.** This figure is updated from Dhimal *et al.* 2014 [1].

A detailed description of Nepal and its administrative and geographic divisions has been provided in a previous publication [1]. Out of 13 high-risk malaria districts in Nepal, one from the eastern development region (Morang) and one from the far-western development region (Kailai) of Nepal were selected for this study. The study areas are shown in Figure 1. These two districts contain a substantial proportion of all malaria cases in the respective regions. According to the 2011 Census of Nepal, the total population of Morang district is 964,709 and that of Kailali district, 775,709 [15]. Morang district has 66 VDCs including one submetropolitan city, Biratnagar. Similarly, Kailali district has 44 VDCs including three municipalities. Although a new classification of VDCs and municipalities was established in 2014, the old classification was used for this study because the available data conform to the old classification. The area of Morang district covers 1,855 sq km and that of Kailali district 3,235 sq km. Both comprise lowlands of the terai, forest and foothills, and extend to the hills. The principal malaria vectors of study areas are *Anopheles fluviatilis*, *Anopheles annularis* and *Anopheles maculatus* complex members [1, 16, 17].

### Research design

A retrospective study was designed to assess the association between malaria incidence, climatic variables and vector control interventions in two high-risk malaria districts of Nepal between 2004 and 2012.

### Data collection

A data collection tool was used to enter the number of houses and population covered by LLINs and IRS in each VDC or municipality. These figures were then submitted to District (Public) Health Office (DPHO) who in turn produced VDC level reports that were latter consolidated at both district and national level for each year. The VDCs were selected for LLIN distribution considering the following criteria [3]:

1. LLINs were provided to population of all wards of high-to-moderate risk VDCs except the population of VDCs protected by IRS.
2. Forecasting of LLINs was done as population projected to be covered/1.8.

Two rounds of IRS were carried out routinely in high-risk VDCs. The first round of IRS (IRS1) was undertaken during pre-monsoon (April-May) and the second round of IRS (IRS2) in monsoon (July-August) each year [1, 3]. In the last five years, synthetic pyrethroid insecticides such as lambda-cyhalothrin (25 mg/m^2^), deltamethrin (20 mg/m^2^) and alpha-cypermethrin (30 mg/m^2^) have been used in rotation for IRS, and susceptibility tests using the WHO acute test procedure showed that all anopheline mosquitoes tested were susceptible to these insecticides [3, 5]. Although LLINs and IRS are complementary interventions, the present policy is to increase the coverage of LLINs and to reduce IRS. During 2007–2008, LLINs were distributed with a policy of one LLIN per household but this changed to one LLIN per two persons in a house in 2009 [1, 3]. In addition, LLINs were also provided free of cost by DPHO to all pregnant women attending antenatal care clinics at the public health institutions. The LLIN distribution strategy is to target one third of VDCs per year in each district and cover all high-risk VDCs by the end of the third year [18]. In GFAMT program districts, LLINs were distributed through mass campaigns. The LLINs distribution started in both districts from 2006 to 2008 by Population Service International (PSI)/Nepal in partnership with local NGO through social marketing. After 2008, social marketing stopped and LLINS were distributed freely by PSI in the GFAMT program areas. The IRS was done by DPHO. The DPHO collected and stored IRS and LLINs data. Finally, these data were reported to Epidemiology and Disease Control Division (EDCD) through health management information system (HMIS). Monthly aggregated district-level malaria data between 2004 and 2012 were obtained from the EDCD, Department of Health Services (DoHS), Ministry of Health and Population (MoHP), Government of Nepal. Similarly, annually aggregated VDC or municipality level (i.e., the smallest administrative unit in each district) malaria indicators and vector control intervention data were also obtained from the EDCD. These data were collected during a malaria microstratification study in 2012–2013. However, VDC level data were compiled only between 2007 and 2011 because the complete data for 2012 were not available at the time of data collection and vector control interventions (mainly distributing LLINs only) started in 2006 in these two districts. The details of the malaria surveillance system in Nepal have been described in a previous publication [1]. The data were verified by personnel of EDCD and cleaned independently by two members of this study team.

All of the malaria cases used in this study were confirmed cases, either by microscopy or RDT kits. Data on the population at risk of malaria at both VDC and district levels were obtained from the EDCD and were projected using national population census data of 2001 and 2011 [15, 19]. Records of both imported and indigenous cases were based on travel history as recorded in the Nepalese HMIS system. Indigenous malaria is defined as “Malaria acquired by mosquito transmission in an area where malaria is a regular occurrence” [20]. Geo-environmental data were collected based on classification of VDCs using topographic maps of the study districts. The monthly accumulated rainfall (mm), air temperature (minimum and maximum) (°C) and average relative humidity (RH) (%) of respective districts were obtained from the Department of Hydrology and Meteorology (DHM), Ministry of Science, Technology and Environment, Government of Nepal.

### Hotspot analysis

Annual malaria incidence at the VDC level was analysed independently for spatial clustering (or hotspots) using the Getis-Ord Gi* statistic [21–23] in the Arc GIS software version 10 (ESRI, Redland, CA, USA). This statistic reflects whether differences between the local mean (i.e., the incidence for a VDC and its nearest neighbouring VDC within a district) was significantly different from the global mean (i.e., the overall incidence of all VDCs for that particular district) [22, 24, 25]. A statistically significant positive z-score value shows a hotspot for high incidence rates while a statistically significant negative z-score value for a VDC specifies local spatial clustering of low incidence rates [22, 24–26]. Two separate cluster analysis was performed and presented with one using all malaria cases and one with just imported malaria cases.

### Statistical analysis

The data were entered in Microsoft Excel and analysed in R computing software [27]. Assuming an average net life of three years and one net per two household residents, VDC level coverage of LLINs were calculated per person [1, 3]. Similarly, VDC level IRS coverage was calculated per person using number of households covered by IRS and population residing in those households. The incidence of malaria was expressed per 10,000 population at risk of malaria. Generalized additive mixed models (GAMM) were used to assess the effects of climatic variables on malaria incidence using the district-level monthly aggregated data, and the effects of vector control interventions on malaria incidence using annually aggregated VDC level data. A GAMM is an extension of generalized linear models that allows random effects to be included in the predictor, and non-parametric smoothing terms in the place of the constant parameters [28, 29].

The use of the generalized additive model (GAM) approach is useful for this study because it provides a flexible method to identify nonlinear covariate effects in exponential family and other likelihood-based regression methods [30]. Furthermore, instead of estimating a single parameter, GAM provides a general unspecified (non-parametric) function that relates the predicted (transformed) response values to the predictor values. The Poisson distribution with a log link function for the effects of climatic factors and vector-control interventions on the malaria incidence was used. As the collected data of malaria, vector control interventions and climate were of different spatial (district and village level) and temporal (annual and monthly) scales, the following two separate models were fitted to the data and can be summarised as:

The random effects are denoted by (1|X). In the first model (Model I), year was used to model variation between districts and months. In the second model (Model II), VDC was used to model variation between year, districts and geo-environmental regions. Similarly, a spline (denoted s(x)) was fitted to assess the association between malaria incidence and climatic factors (monthly data) and vector control interventions (IRS and LLINs) using the annually aggregated data. Climatic factors and vector control interventions seem to have linear effects as their effective degrees of freedom (‘edf’) were estimated as 1, which indicates that the spline is not distinguishable from a straight line. In order to assess changes in malaria incidence, risk ratios (RR) were calculated to observe differences in means between discrete time periods or places [31]. The log of the malaria incidence rate is expected to be linearly associated with the vector control interventions or climatic variables of different time periods, and the model parameters after exponentiation can be interpreted as RR, which is similar to relative risk or relative incidence [1, 32]. Models were fitted in R using its ‘mgcv’ package [28].

### Ethical considerations

The Ethical Review Board of the Nepal Health Research Council (NHRC) approved the conduct of this study. Only data that had been approved and documented by the EDCD, DoHS, Government of Nepal, were used for this study.

## Results

### Changes in the burden of malaria in two malaria high-risk districts

Opposing trends in malaria incidences were observed in the districts after the introduction of LLINs (Table 1). Confirmed malaria incidence was reduced from 2.24 per 10,000 in 2007 to 0.31 per 10,000 population in the Morang district of eastern Nepal. In contrast, confirmed malaria incidence increased from 3.38 per 10,000 to 8.29 per 10,000 population in Kailali district of far-western Nepal. More than 50% of the cases in Kailali district were attributed to imported malaria cases. The proportion of both indigenous malaria cases in general and that of indigenous *P. falciparum* cases were higher in Morang than Kailali district. The LLINs and first IRS coverage were higher in Kailali district compared to Morang district. The incidence of malaria was 82% lower in Morang than in Kailali district (RR = 0.18, 95% CI = 0.11-0.33).

**Table 1:** **Reported confirmed malaria cases and vector control interventions in two high-risk districts in Nepal (2007–2011)**

Year	District	2007	2008	2009	2010	2011	Mean
Confirmed malaria	Kailali	3.38	3.83	5.96	9.03	8.29	6.10
Incidence/10,000 persons	Morang	2.24	2.07	1.02	0.76	0.31	1.28
Indigenous malaria	Kailali	1.62	1.88	3.16	4.59	3.73	3.00
Incidence/10,000 persons	Morang	1.79	1.88	0.77	0.42	0.19	1.01
Malaria incidence <5 year	Kailai	0.33	0.20	0.72	2.87	0.44	0.91
/10,000 persons	Morang	0.80	1.03	0.50	0.05	0.08	0.49
Malaria incidence >5 year	Kailai	3.96	4.52	6.75	9.92	9.38	6.91
/10,000 persons	Morang	2.41	2.20	1.09	0.84	0.37	1.38
LLINS coverage	Kailai	11.13	109.78	254.86	454.52	301.51	226.36
/1,000 population	Morang	30.40	29.74	69.20	108.14	111.10	69.72
First round IRS coverage/	Kailai	141.21	100.14	55.16	19.29	11.25	65.41
1,000 persons	Morang	28.16	7.70	26.06	0.00	13.14	15.01
Second round IRS coverage	Kailai	30.50	21.35	4.33	14.72	8.40	15.86
/1,000 persons	Morang	36.88	61.73	10.71	17.93	0.00	25.45
% of indigenous	Kailai	9.49	13.88	13.47	7.75	3.38	9.59
*P. falciparum*	Morang	31.88	44.63	45.05	24.05	21.62	33.44
% indigenous *P. vivax*	Kailai	36.39	35.89	35.24	42.00	41.22	38.96
	Morang	53.36	43.00	35.14	69.62	43.24	45.67
% Indigenous cases	Kailai	45.89	49.76	48.71	49.75	44.59	47.74
	Mornag	88.26	92.18	85.59	69.62	64.86	80.10
% Imported cases	Kailai	54.11	50.24	51.29	50.25	55.41	52.24
	Morang	11.74	7.82	14.41	30.38	35.14	13.46
Confirmation by microscopy ( %)	Kailai	100	60.05	64.58	57.50	57.84	64.10
	Mornag	100	100	75.68	84.81	100	95.31
Confirmation by RDT (%)	Kailai	0	39.95	35.42	42.50	42.16	35.90
	Mornag	0	0	24.32	15.19	0	4.69

### Hotspots

**Figure 2:**
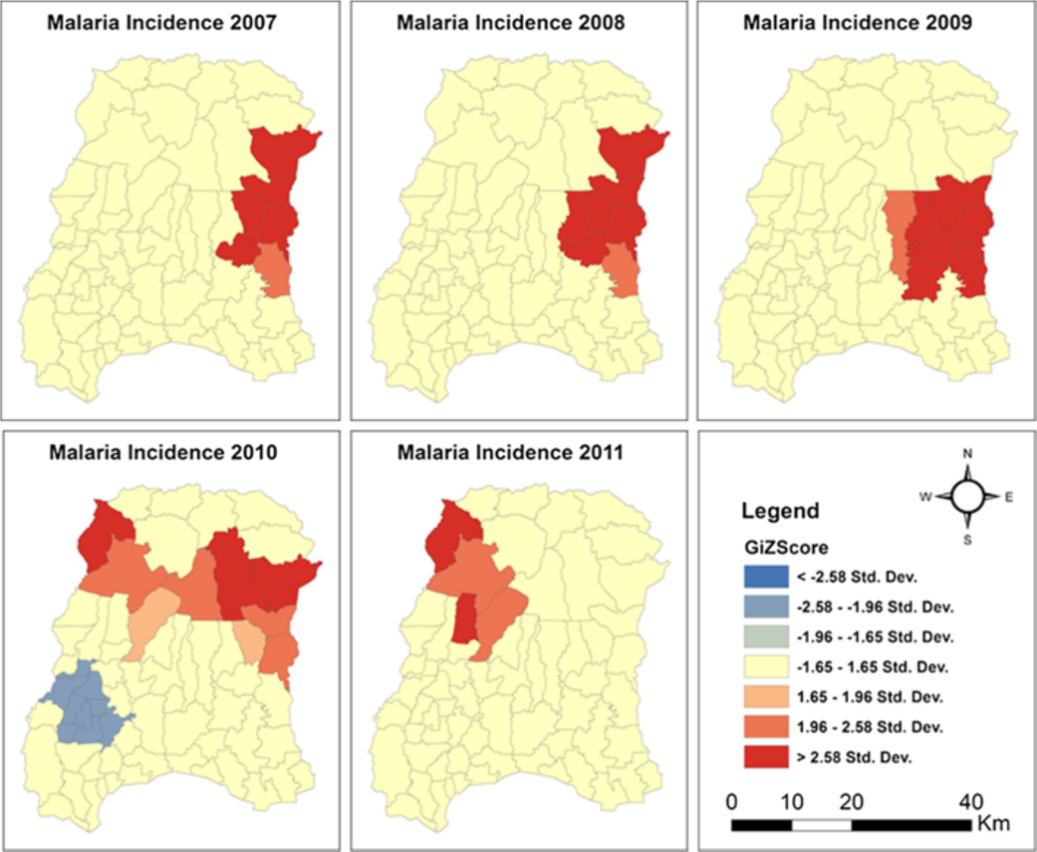
**Confirmed malaria hotspots in Morang district (2007–2011).**

**Figure 3:**
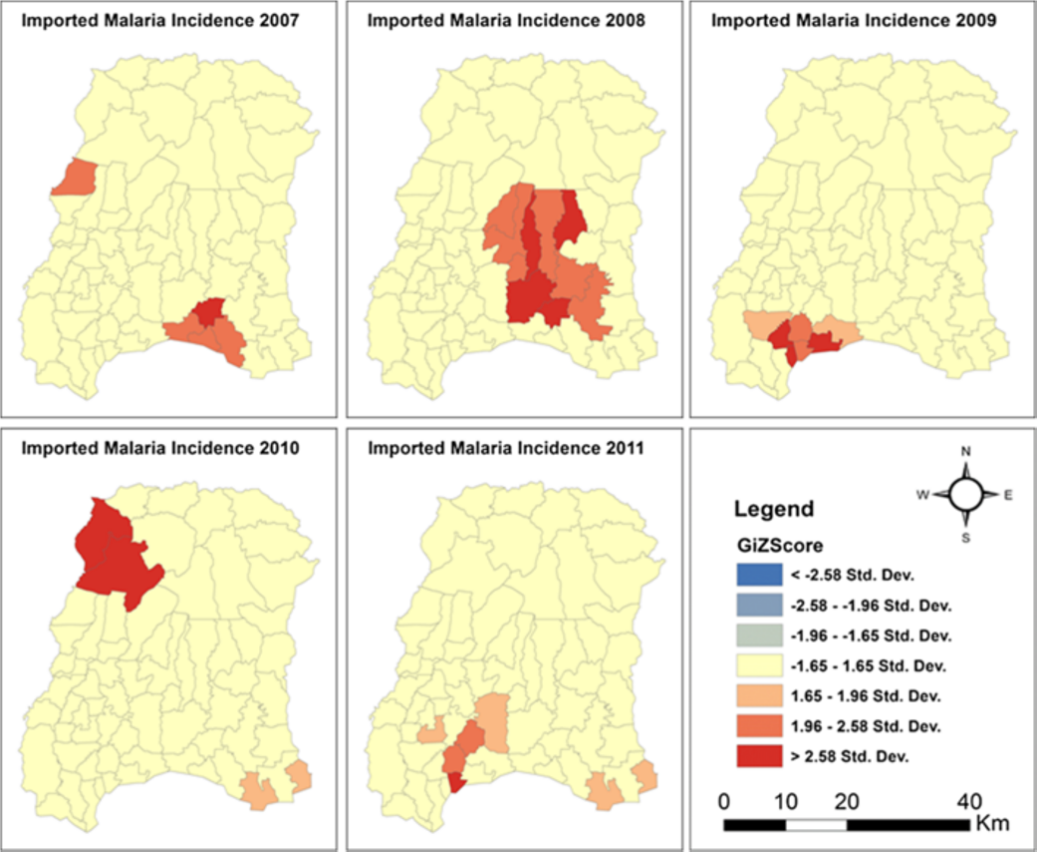
**Imported malaria hotspots in Morang district (2007–2011).**

**Figure 4:**
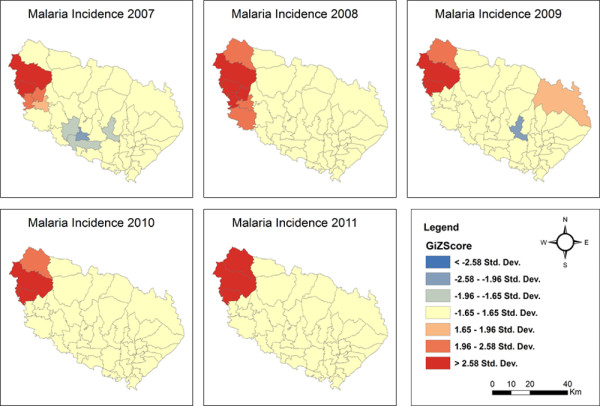
**Confirmed malaria hotspots in Kailali district (2007–2011).**

**Figure 5:**
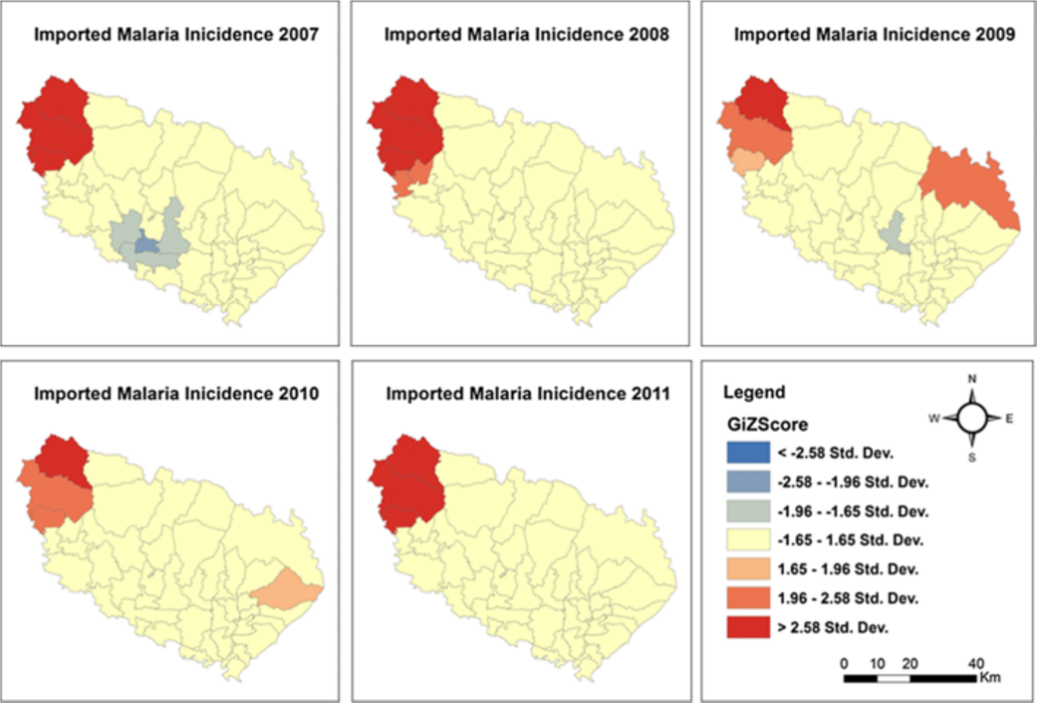
**Imported malaria hotspots in Kailali district (2007–2011).**

Applying hotspot analysis to malaria incidence data showed that significant malaria hotspots (*P* <0.01, z > 2.58) were present at the VDC level in both districts. In Morang district, significant malaria hotspots (all malaria cases) shifted over the years from the eastern to the north-western part of the district as shown in Figure 2. The VDCs with new hotspots in 2011 were Belbari and Indrapur (terai plains), and Kerbari and Yangsila (forest, forest fringe and foothills) of Morang dstrict. Similarly, imported malaria hotspots also shifted over the years in new VDCs (Figure 3). In contrast, despite vector control interventions, significant malaria hotspots persisted in the same VDCs over the years, mainly in Malakheti, Godwari and Sahajpur (forest, forest fringe and foothill regions) of Kailali district which are located in the western part of the district neighbouring Kanchanpur district (Figures 4 and 5).

### Associations between climatic factors and the burden of malaria

**Figure 6:**
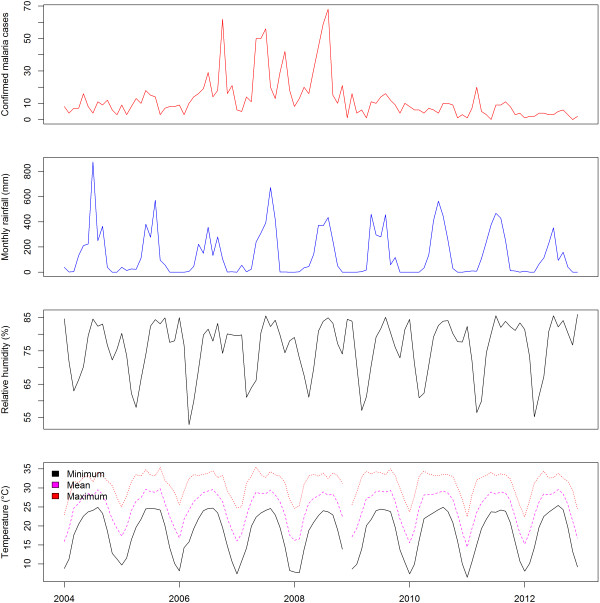
**Monthly malaria cases of Morang district with temperature, relative humidity and rainfall from 2004 to 2012.**

**Figure 7:**
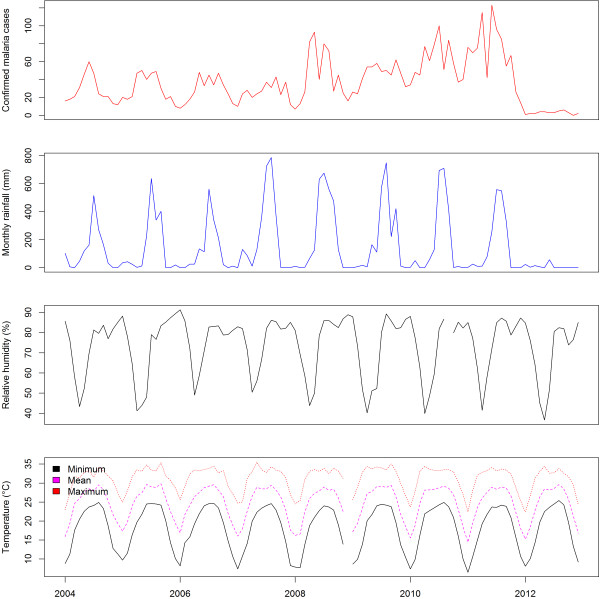
**Monthly malaria cases of Kailali district with temperature, relative humidity and rainfall from 2004 to 2012.**

**Figure 8:**
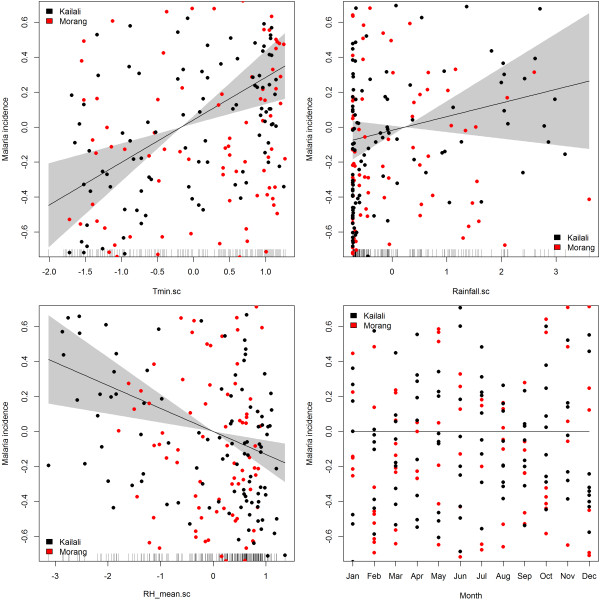
**Effects of climatic factors and month on malaria incidence in Morang and Kailali districts (2004 to 2012).** The solid line is the estimated effect, grey polygon is the 95% confidence region. The y-axis of each plot represents risk ratio of malaria incidence in log scale.

The minimum temperature, mean temperature and average RH were linearly associated with malaria incidence. The time series of monthly malaria cases of Morang and Kailaili district with temperature, humidity and rainfall are shown in Figures 6 and 7, respectively. Among the climatic variables only minimum temperature and RH were significant predictors of malaria incidence (Figure 8). Overall, a 1°C increase in minimum temperature increased malaria incidence by 27% (RR = 1.27, 95% CI = 1.12-1.45) and a 1% increase in mean RH decreased malaria incidence by 9% (RR = 0.91, 95% CI = 0.83-1.00). However, when mean temperature was used in model instead of minimum temperature, only mean temperature was a significant predictor. Overall, a 1°C increase in temperature increased malaria incidence by 25% (RR = 1.25, 95% CI = 1.11-1.43) (see Additional file 1). The effects of maximum temperature and rainfall on malaria incidence were not significant.

### Associations between vector control interventions and the burden of malaria

**Figure 9:**
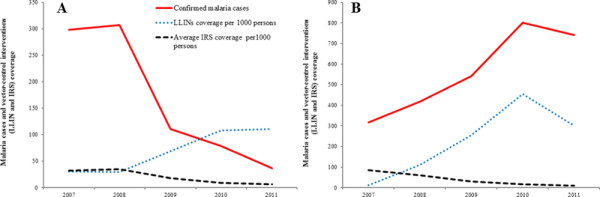
**Confirmed malaria cases and vector-control interventions in Morang and Kailali districts (2007–2011).** Panel **A** shows confirmed malaria cases, LLINs and average IRS coverage of Morang district. Panel **B** shows confirmed malaria cases, LLINs and average IRS coverage of Kailali district.

**Figure 10:**
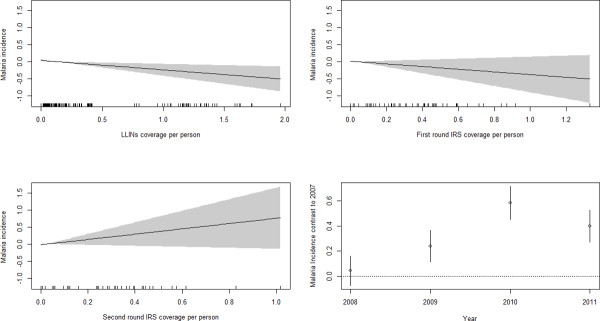
**Effects of vector-control interventions and year on malaria incidence in Mornag and Kailali district (2007–2011).** The y-axis of each plot represents risk ratio of malaria incidence in log scale.

The number of confirmed malaria cases and vector-control interventions (average IRS and LLINs coverage) in Morang and Kailai district is shown in Figures 9A and B, respectively. The effects of vector-control interventions and year on malaria incidence are presented in Figure 10. The decline in the burden of malaria was associated with LLIN coverage at the VDC level. Malaria incidence was reduced by 25% per one unit increase of LLINs (RR = 0.75, 95% CI = 0.62-0.92).The effect of both rounds of IRS was not significantly associated with malaria incidence. The combined effect of differences in changes in intervention over time shows significant effect of year on malaria incidence. The incidence of s malaria was significantly higher in 2010 (RR = 1.79, 95% CI = 1.38-2.32) and in 2011 (RR = 1.48, 95% CI = 1.16-1.9) compared to 2007. The incidence of malaria was significantly lower in hills and river valleys compared to plain *terai* (RR = 0.29, RR = 0.11-0.76). Although statistically not significant, the incidence of malaria was significantly higher in forest, forest fringe and foothills compared to plain *terai* (RR = 1.68, 95% CI = 0.90 – 3.11).

## Discussion

Nepal has prepared for malaria pre-elimination since 2011 with the ambitious goal of malaria elimination by 2026. However, shifts of malaria hotspots to new VDCs in Morang district and stable malaria hotspots persist in specific VDCs of Kailali district, despite more than six years of continuous vector control interventions using IRS and LLINs, indicates that there are problems with the malaria elimination efforts in the country.

Despite declining trends of malaria in Morang district, malaria hotspots have shifted to new VDCs, which in the past had been regarded as low-risk VDCs so that vector control interventions were not in place. The hotspots were reported in eastern villages in 2009 since LLINs started to be distributed in high endemic VDCs of Morang district with one LLIN per two household persons policy in 2009. Malaria spread west in 2010 because eastern village hotspots disappeared and gradually shifted into forest and hill areas of the western part of the district. In contrast, despite vector control interventions since 2006, hotspots remained in the same VDCs in Kailali district. Both districts share a border with high-endemic districts in Nepal as well as with India. The scaling-up of LLINs started in Kailali and Morang districts in 2006. However, IRS activity was regular before and during the study period. Only LLINs is found to be associated with a decline of malaria incidence in the present study, which is consistent with findings from Bangladesh [24] Rwanda [33], and Zambia [34]. In contrast to findings of this study, malaria incidence was associated with IRS coverage only in Botswana [22]. These findings imply that vector control interventions with a low coverage, or those that are focussed on selected VDCs only, cannot break down malaria transmission in VDCs or districts bordering malaria-endemic areas because the presence of vectors, a suitable climate and the continuous import of malaria cases facilitate the transmission cycle. Furthermore, the higher incidence of malaria in the forest, forest fringe and foothills, especially in the high-risk VDCs of Kailali district where regular movement of people is high, indicates that forest-related malaria cannot be controlled using vector control interventions such as LLINs and IRS alone. For this, effective BCC strategies are needed to promote avoiding mosquito-man contact, e.g., by the use of mosquito repellents and/or protective clothing.

The proportion of imported malaria cases has almost constant over the years in both districts, consisting of more than 50% in Kailali district and 30% in Morang district (Table 1), which is consistent with previous findings [1]. Imported malaria has appeared as a major challenge for many countries embarking on malaria elimination [22, 35–37]. Large-scale migration within and outside the district, across the southern border of Nepal with India, displacement of the population after natural disasters such as floods, and the continuous introduction of malaria cases among adult migrant workers could be seen as factors for the persistence of malaria, especially in Kailali district. Hence, a cross-border malaria strategy that is well coordinated with malaria-endemic neighbouring counties such as India is very urgently needed. In the absence of the implementation of such a strategy, regular border screening among travellers should be carried out by establishing check posts for acute febrile illness at the border crossings, which may contribute to a reduction in malaria transmission among other benefits.

Climatic factors can be important for increasing the risk of malaria transmission especially in sub-tropical and temperate regions where the minimum temperature is a limiting factor for malaria transmission. The positive association of minimum and mean temperature with malaria incidence in the present study is consistent with the findings of many previous studies [12–14, 38–45]. An increase in the minimum (and also mean) temperature increases mosquito abundance and biting rates and shortens the incubation period of *Plasmodium* parasites, thereby increasing the malaria transmission cycle. Minimum temperature is the most influential environmental variable for malaria transmission since it occurs at night [46]. This is because malaria vector mosquitoes remain active for biting during the night when people sleeping without bed nets are exposed to the bite of infected mosquitoes, resulting malaria infection. Furthermore, when exposed to high temperatures at night people usually do not cover themselves and some people (usually adult men) sleep outside the house under the trees to avoid the heat, which in turn increases the risk of malaria [46–48]. These observations are consistent with the findings of this study that the incidence of malaria was higher among adults. In contrast, maximum temperature has a complex relationship to malaria because an increase in maximum temperature above a certain range interrupts mosquito and parasite development [8, 14]. Similarly, the effect of rainfall on malaria incidence is complex. In areas where their breeding sites are produced by rainfall, increasing rainfall increases mosquito populations. However, too little rain, or drought, affects the mosquito life cycle as well as too much rainfall which can flush away the breeding places and thus decrease mosquito abundance [11, 49–51].

No significant effect of the month of the year on malaria incidence was observed, which indicates a perennial distribution of malaria in both districts. Thus, IRS spraying in the pre-monsoon (April-May) and monsoon (July-August) seasons only may be ineffective for controlling malaria vectors. This observation can be explained by the fact that the minimum temperature increased rapidly in the terai region in all seasons [52, 53], so that the transmission of malaria was possible even in winter. In contrast, an increase in mean temperature above 28°C [14] mainly influenced by maximum temperature drastically reduces malaria transmission, particularly in the summer, which is the warmest season in Nepal. As a result of these two contrasting effects, coupled with an influx of infected migrant workers in the post-monsoon and winter seasons, the effect of the month on malaria incidence may be insignificant in this setting. Alternatively, there might be no effect of the month because temperature alone is sufficient to explain seasonal variation. Based on the association found between malaria incidence and temperature, malaria transmission in the temperate regions of Nepal can be predicted as revealed elsewhere by many previous studies [12–14, 45].

The latest microstratification of malaria at the VDC level may be very useful for an effective utilization of limited resources. However, malaria microstratification should be updated regularly according to the progress with an aim to target new hotspots. As the country progresses towards elimination, finer scale mapping, i.e., at the ward or household level, is needed to identify residual foci [54–56]. For example, a study from Bangladesh identifies stable malaria hotspots and risk factors at the household level which guide for cost-effective targeting of malaria intervention that may finally contribute to potential elimination of malaria from the country [51]. The application of spatial decision support tools such as geographic position system (GPS), geographical information system (GIS) and mobile computing technology helps to identify spatial clusters of malaria transmission and provides effective monitoring, evaluation and surveillance tools to cope with the complexities that are associated with the spatial variability of malaria transmission and associated risk factors [57].

This study, like other studies that use secondary data, has several limitations so the findings should be interpreted with caution. First, the analyses were based on routinely collected passive surveillance data from public health institutions only. As reported elsewhere [2], malaria cases reported to the EDCD and malaria cases reported through the (HMIS) vary slightly. Monthly malaria data used in this study were aggregated at the district level and collected through the HMIS while yearly aggregated VDC-level malaria and vector control intervention data were collected from visits to each health institution of a district and from the EDCD, which resulted in slight differences in data in some years. Second, the observed association between malaria incidence with LLINs and IRS coverage and climatic factors is ecological and not at the level of individuals. Third, malaria incidence was calculated based on confirmed malaria cases at public health institutions only and therefore misses out on possible cases from the private healthcare sector. As a result, the results of this study may not represent the situation of malaria transmission at the population level. Furthermore, a single model could not be developed since the collected data of malaria, vector control interventions and climate factors were of different spatial (district and village level) and temporal (annual and monthly) scales. Despite these challenges, this study provides important information about the malaria situation at district and subdistrict (VDC) levels after the scaling-up of malaria control interventions from the GFAMT support, and this will be important for preparing the malaria pre-elimination phase in Nepal.

## Conclusions

Despite a significant decline in malaria cases at the national level, an increasing trend of malaria incidence in Kailali district, with persistence of malaria cases in the same villages where vector control interventions had been in place, a shift of malaria hotspots to new villages in Morang district without vector control interventions, and a positive association of malaria incidence with temperature indicates worries about the elimination of malaria from the country. However, the malaria elimination goal can be achieved if hotspots of malaria can be identified accurately and vector control interventions such as LLIN coverage can be scaled up in the entire endemic districts rather than focusing only on selected VDCs. This is important because the movement of people within and between districts and across the border to India is high. Second, imported malaria cases should be controlled by establishing health check posts at the border crossings screening for people with acute febrile illness. Third, community-based prevalence surveys should be carried out to detect asymptomatic malaria cases, identify spatial clusters of malaria hotspots and determine the real malaria transmission situation at the population level. Fourth, data recording, reporting and surveillance systems should be strengthened and a case-based surveillance system should be started in all malaria-endemic districts. Fifth, microstratification of malaria transmission areas should be carried out integrating observed climatic data and high resolution remote sensing images, and differential diagnosis of any fever case coming from probable malaria transmission areas should be performed. Finally, continuous efforts are crucial to maintain and sustain the gains that have already been achieved.

## Electronic supplementary material

Additional file 1:**Effect of mean temperature on malaria incidence (2004–2012).**(TIFF 68 KB)
