# Supplementary figures and images for: Spatio-temporal distribution of malaria and its association with climatic factors and vector-control interventions in two high-risk districts of Nepal
Source: Malar J. 2014 Nov 25;13:457. doi: 10.1186/1475-2875-13-457 (PMC4258811; doi:10.1186/1475-2875-13-457)

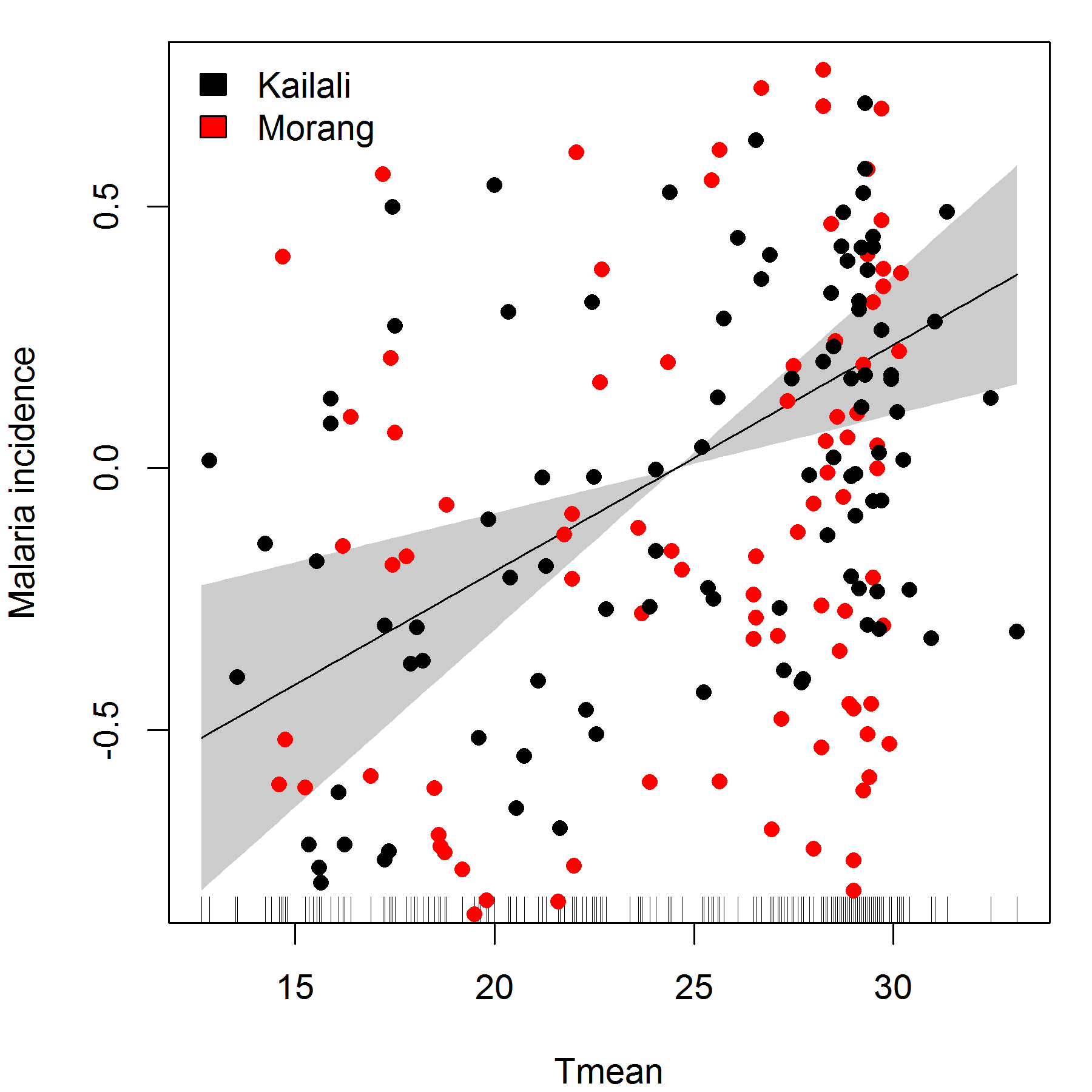

Supplement: Supplementary file 1 — Additional file 1:Effect of mean temperature on malaria incidence (2004–2012).(TIFF 68 KB) [file 12936_2014_3616_MOESM1_ESM.tiff]
